# Protective Effects of Herbal, Woody and Lianoid Plant Polysaccharides Against Diabetic Nephropathy: A Review

**DOI:** 10.3390/nu18203362

**Published:** 2026-10-14

**Authors:** Wudan Cai, Shanshan Jin, Ziming Huang, Huan Liu, Qin Wu, Xiaoyue Xiao, Yongshu Li, Jianjun Hou, Xixia Liu, Mingcong Fan

**Affiliations:** 1 Hubei Key Laboratory of Edible Wild Plants Conservation & Utilization, College of Life Sciences, Hubei Normal University, Huangshi 435002, China; 1571207030@qq.com (Z.H.); liuhuanjzcd@126.com (H.L.); 397875957@qq.com (Q.W.); xiaoyuexiao@hbnu.edu.cn (X.X.); yongshuli@hbnu.edu.cn (Y.L.); 340407758@qq.com (J.H.); liuxixia@hbnu.edu.cn (X.L.); 2 Hubei Engineering Research Center of Special Wild Vegetables Breeding and Comprehensive Utilization Technology, Hubei Normal University, Huangshi 435002, China; 3 Future Food and Agriculture Department, Yangtze Delta Region Institute of Tsinghua University, Jiaxing 314006, China; majicjs@163.com

**Keywords:** plant polysaccharides, diabetic nephropathy, oxidative stress, inflammation, renal fibrosis, gut microbiota

## Abstract

Diabetic nephropathy (DN) is one of the most severe microvascular complications of diabetes mellitus and has become the leading cause of end-stage renal disease. Natural plant polysaccharides are bioactive natural macromolecular compounds widely distributed in medicinal plants, fruits, vegetables and cereals. Owing to their multi-target effects and low toxicity, they have attracted considerable attention in the prevention and treatment of DN. Numerous studies have shown that plant polysaccharides may exert renal protective effects through various mechanisms, including antioxidative stress, anti-inflammation, anti-fibrosis, podocyte protection, inhibition of advanced glycation end-product formation, and regulation of the gut microbiota. This paper systematically reviews recent research advances concerning plant polysaccharides for DN intervention, with an emphasis on their action mechanisms, and discusses the structure–activity relationships. However, there are still challenges facing the clinical translation of polysaccharides, including low bioavailability and difficulties in quality control. Future research should focus on the precise analysis of the advanced structure of polysaccharides, clarification of the mechanism of action based on the intestinal microecology, and the development of new delivery systems to promote the clinical application of plant polysaccharides in DN treatment.

## 1. Introduction

With substantial changes in global lifestyles and dietary patterns, the incidence of diabetes has exhibited a progressive upward trend with increasing onset at younger ages, constituting a major public health challenge associated with chronic metabolic diseases worldwide. Diabetic nephropathy (DN) is one of the most common and severe microvascular complications of diabetes and represents the leading cause of end-stage renal disease [1]. Globally, approximately 20–40% of patients with type 2 diabetes mellitus will progressively develop DN, ultimately resulting in irreversible renal function decline that necessitates dependence on dialysis or kidney transplantation for survival [2]. This not only severely compromises patients’ quality of life but also imposes a substantial economic burden on families and healthcare systems.

**Figure 1:**
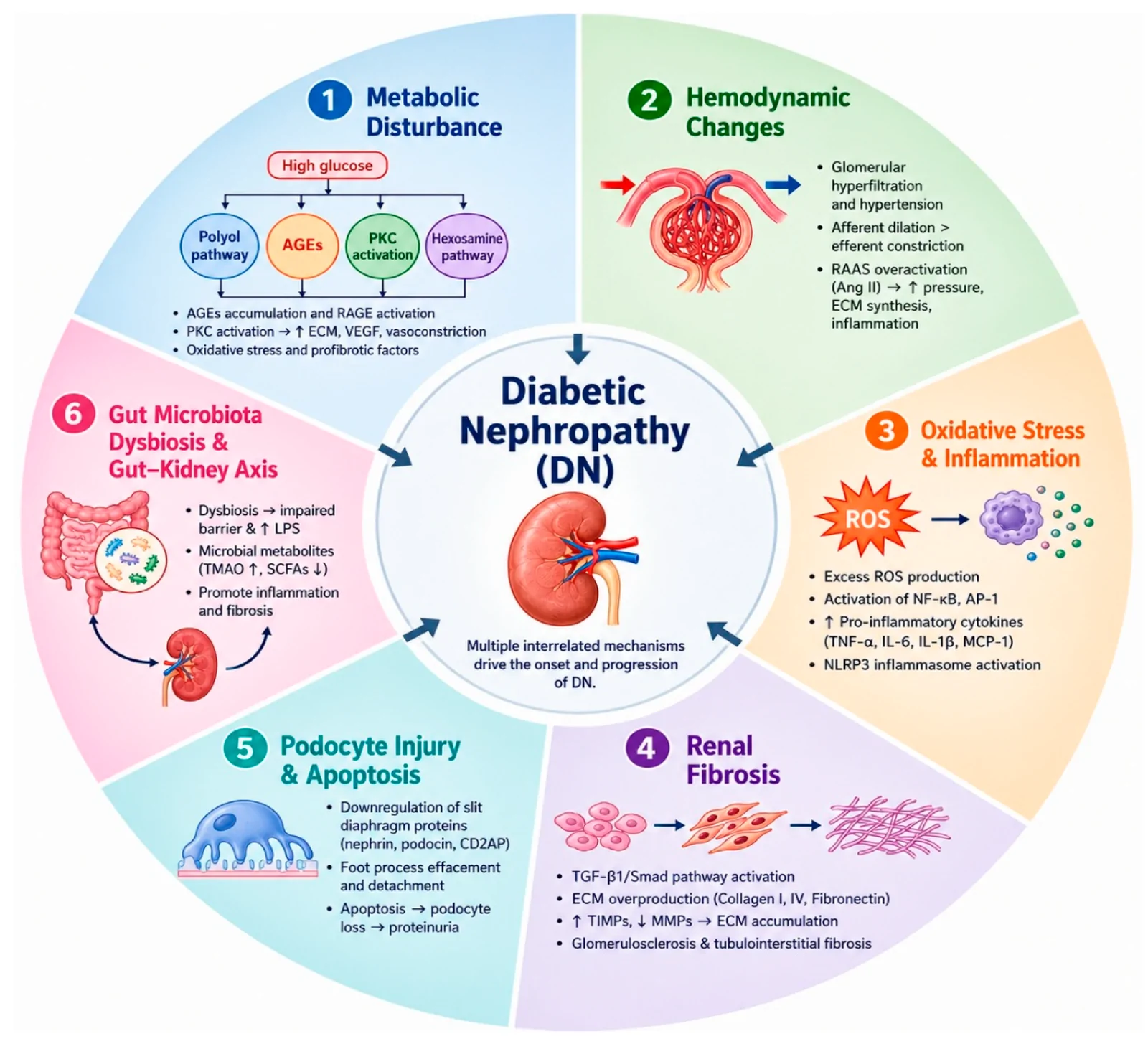
The core pathogenesis of DN.

The principal pathological hallmarks of DN include markedly increased urinary albumin excretion, expansion of the glomerular mesangial area, thickening of the basement membrane, and renal tubulointerstitial fibrosis [3]. Its pathogenesis is closely associated with multiple factors, including glucolipid metabolic disorders, hemodynamic abnormalities, oxidative stress, and inflammatory responses (Figure 1) [4]. Currently, clinical therapeutic options for DN remain relatively limited, predominantly centered on glycemic, blood pressure, and lipid control, along with lifestyle interventions [5]. Pharmacological management of DN has evolved into a multi-target, guideline-directed “four-pillar” strategy. First, renin–angiotensin system inhibitors, lower intraglomerular pressure and reduce albuminuria. Second, sodium-glucose cotransporter-2 inhibitors slow chronic kidney disease (CKD) progression, reduce the risk of acute kidney injury and cardiovascular events, and are recommended for patients with type 2 diabetes, CKD, and an estimated glomerular filtration rate of at least 20 mL/min/1.73 m^2^. Third, glucagon-like peptide-1 receptor agonists improve glycemic control and confer additional anti-inflammatory and renal protective effects. Fourth, nonsteroidal mineralocorticoid receptor antagonists such as finerenone attenuate mineralocorticoid receptor overactivation, albuminuria, inflammation, and fibrosis [6]. Although these therapeutic strategies can delay the progression of DN to a certain extent, they fail to fundamentally reverse pathological damage to renal tissue. Furthermore, long-term administration is associated with issues such as drug resistance, hepatorenal toxicity, and increased adverse effects [7]. Consequently, the development of novel therapeutic agents with multi-target regulatory effects and favorable safety profiles has become a prominent research focus in the prevention and treatment of DN.

Plant polysaccharides (PPS) are high-molecular-weight polymers composed of more than ten monosaccharide units linked by glycosidic bonds. Owing to their abundant sources, favorable safety profiles, diverse biological activities, and minimal toxic side effects, they exhibit broad application prospects for the prevention and adjunctive treatment of DN based on existing preclinical evidence [8]. Beyond pharmacological candidates, PPS represent an important component of the human diet. Many of these compounds function as dietary fibers and bioactive constituents, exerting systemic benefits via metabolic improvement, gut-homeostasis support, and immunomodulation, which may indirectly contribute to kidney-protective outcomes. For example, the Moutan cortex polysaccharide not only remodels the gut microbiota, improves intestinal barrier function, and elevates the content of short-chain fatty acids but also attenuates hyperglycemia and renal injury in DN rats by reducing serum levels of pro-inflammatory mediators [9]. *Bupleurum* polysaccharide inhibits renal inflammation and fibrosis progression in diabetic mice through the suppression of the high-mobility group box 1 (HMGB1)–toll-like receptor 4 (TLR4) pathway [10]. *Astragalus mongholicus* polysaccharide alleviates oxidative damage and inflammatory responses in DN via activation of the nuclear factor erythroid 2-related factor 2 (Nrf-2)/Kelch-like ECH-associated protein 1 (Keap1) pathway and inhibition of the TLR4/nuclear factor kappa B (NF-κB) pathway, while also ameliorating renal fibrosis progression through regulation of the transforming growth factor beta (TGF-β)/Smad family member 4 (Smad4) signaling pathway [11]. Although numerous studies have demonstrated that PPS can effectively mitigate renal pathological damage and retard renal function decline in preclinical DN cell and animal models, current research still faces certain limitations, including insufficient in-depth mechanistic elucidation, unclear structure–activity relationships, and a paucity of clinical investigations.

Therefore, this review systematically elaborates on recent research advances regarding the intervention of PPS in DN, with particular emphasis on summarizing the underlying mechanisms and structure–activity relationships. Furthermore, future research directions and prospects for clinical translation are discussed. This review aims to provide a theoretical reference for basic research, novel drug development, and potential clinical adjunctive therapy of PPS in the context of DN.

## 2. Sources and Physicochemical Properties of PPS

PPS are widely distributed in the roots, stems, leaves, flowers, fruits, and seeds of various plants. To date, PPS reported to exhibit anti-DN activity can be categorized according to their botanical sources into herbal, woody, and lianoid. PPS with anti-DN activity derived from different sources have been summarized in Table 1.

Numerous studies have demonstrated that PPS exhibit a wide range of biological activities, including antioxidant, hypoglycemic and hypolipidemic effects, and immunomodulatory properties, which can effectively alleviate or inhibit the progression of DN [12,13]. The bioactivities of PPS are closely associated with their structural characteristics, and polysaccharides from different plant sources possess unique structural features. In terms of the monosaccharide composition, the majority of polysaccharides with renoprotective effects are heteropolysaccharides, primarily composed of various monosaccharides, such as glucose (Glc), galactose (Gal), arabinose (Ara), mannose (Man), xylose (Xyl), and rhamnose (Rha), in varying proportions and linked through diverse glycosidic linkages [14,15]. In particular, the polysaccharide from *Dipsacus asper* wall consists only of D-glucan [16]. Furthermore, available studies have demonstrated that PPS with anti-DN activity exhibited a broad molecular-weight distribution, ranging from 3.9 kDa to 836 kDa (Table 1). In general, a moderate molecular weight may be more conducive to the binding of polysaccharides to target receptors, as well as their absorption and utilization in vivo.

**Table 1:** The sources, molecular weights, monosaccharide compositions and main findings on PPS with DN protection effects.

Category	Source	Polysaccharide Preparation	Molecular Weight (kDa)	Monosaccharide Composition	Model	Major Findings	Reference
Herbal	*Astragalus membranaceus*	Crude polysaccharide			Mouse podocytes; Sprague–Dawley rats	The alleviation of diabetic nephropathy was attributed to the reduction in podocyte apoptosis and the enhancement of autophagy.	[17]
	*Polygonati Rhizoma*	Crude polysaccharide	836 (79.59%)	Fuc, Rha, Glc, Man (1.00:1.28:1.21:0.20)	C57BL/6^db/db^ mice	The renal dysfunction and fibrosis in db/db mice were improved by inhibiting the TGFβ/Smad2 signaling pathway.	[18]
	*Pseudostellaria heterophylla*	Purified polysaccharide fraction			DBA/2 mice; mouse podocytes (MPC-5)	H-1-2 improved high glucose-induced podocyte dysfunction by restoring the activity of SIRT1 and inhibiting the epithelial–mesenchymal transition.	[19]
	*Liriope spicata* var. *prolifera*	Purified polysaccharide fraction	3.9	Fru, Glc (20.7:1)	Wister male rats	LSP downregulated the expression levels of advanced glycation end products and their receptor signaling pathways to alleviate oxidative-stress damage.	[20]
	*Dendrobium officinale* Kimura & Migo	Purified polysaccharide fraction			db/db mice; human renal tubular epithelial cells (HK-2)	DOP alleviated renal fibrosis by inhibiting miRNA-34a-5p/SIRT1 signaling pathway.	[21]
	*Bupleurum*	Crude polysaccharide		Ara, Gal, Glc, Rha (6.35:3.15:1.47:1)	C57BL/6 mice	Mice with DN were protected by BPs via regulating gut microbiota and inhibiting inflammation.	[10]
	*Dipsacus asper*	Purified polysaccharide fraction	89.6	only D-glucose	Wistar rats	DAP achieved its effects by lowering blood sugar, reducing blood lipids, exerting antioxidant functions, and inhibiting the accumulation of AGEs and the expression of RAGE.	[16]
	*Angelica sinensis*	Purified polysaccharide fraction			Sprague–Dawley rats	AG inhibited the survival of GMCs through the RAGE/NF-κB signaling pathway.	[22]
	Celery	Crude polysaccharide			Wistar rats	*Eubacterium rectale* combined with FCP had a synergistic protective effect on diabetic kidney injury.	[23]
	*Momordica charantia*	Crude polysaccharide			Albino rats	MCP inhibited oxidative stress and regulated the HO-1/Nrf2 pathways to alleviate the progression of DN.	[24]
	*Abelmoschus esculentus* (L.) Moench	Purified polysaccharide fraction	626		C57BL/6 mice	Op inhibited cell apoptosis and oxidative stress by activating the AMPK-Sirt1-PGC-1α signaling pathway.	[25]
	Black onion	Crude polysaccharide	8.383 (71.86%)	Man, Rha, GalUA, Glc, Gal, Xyl, Ara (2.67:2.17:5.11:10.77:0.59:72.79:5.91)	Sprague–Dawley rats	The synergistic effect of hypoglycemic and antioxidant actions alleviated liver and kidney function damage.	[14]
	*Andrographis paniculata*	Purified polysaccharide fraction	46	Man, Fuc, Ara, Rha (15.4:2.5:4.3:1.5:1.6)	Wistar rats	The combination of APP and aesculin improved the metabolic abnormalities in diabetic mice and prevented the progression of DN.	[26]
	*Arctium lappa* L.	Purified polysaccharide fraction			C57BL/6J mice	BFO protected the renal function of DN mice by reducing oxidative-stress damage and inhibiting cell apoptosis.	[27]
	Red kidney beans	Purified polysaccharide fraction			Wistar rats	RK and SB exerted anti-diabetic nephropathy effects by regulating the kidney index and influencing key urinary metabolites.	[28]
	Small black soybean	Purified polysaccharide fraction			Wistar rats		[28]
Woody	*Lycium barbarum*	Crude polysaccharide			White rabbits	LBP improved the renal function of diabetic rabbits and alleviated the inflammatory response in the kidneys.	[13]
	*Hippophae rhamnoides* L.	Purified polysaccharide fraction	275.42	Man, Ara, Glc, Gal, Rha (2.02:1.02:4.24:1:9.22)	C57BL/6 mice	SP alleviated DN by reducing renal fibrosis and inhibiting the EMT process.	[29]
	*Camellia oleifera*	Crude polysaccharide			C57BL/6 mice	CFFP improved the renal function of diabetic mice by enhancing antioxidant capacity, reducing lipid peroxidation, inhibiting inflammation and minimizing fibrosis.	[30]
	*Moutan Cortex*	Purified polysaccharide fraction	164	Glc, Ara (3.31:2.25)	Human umbilical vein endothelial cells; Sprague–Dawley rats	MC-Pa exerted its preventive and therapeutic effects on DN by enhancing antioxidant capacity and inhibiting the thickening of the glomerular basement membrane.	[12]
	*Ribes nigrum* L.	Purified polysaccharide fraction	240.508	Man, Rha, GlcA, GalAc, Glc, Gal, Ara (5.09:1.76:3.00:44.15:14.40:17.10:14.50)	C57BL/6J mice	BCP improved renal injury in mice by regulating the intestinal microbiota and influencing bile acid metabolism.	[15]
Lianoid	*Siraitia grosvenorii*	Crude polysaccharide	19.037	Ara, Rib, GalAc, Gal, Man, Glc (1.00:1.72:2.24:3.64:3.89:22.77)	Kunming mice	SGP-1-1 exerted protective effects on DN mice by regulating inflammatory and oxidative-stress responses.	[31]

## 3. Mechanisms of Anti-DN Activity

**Figure 2:**
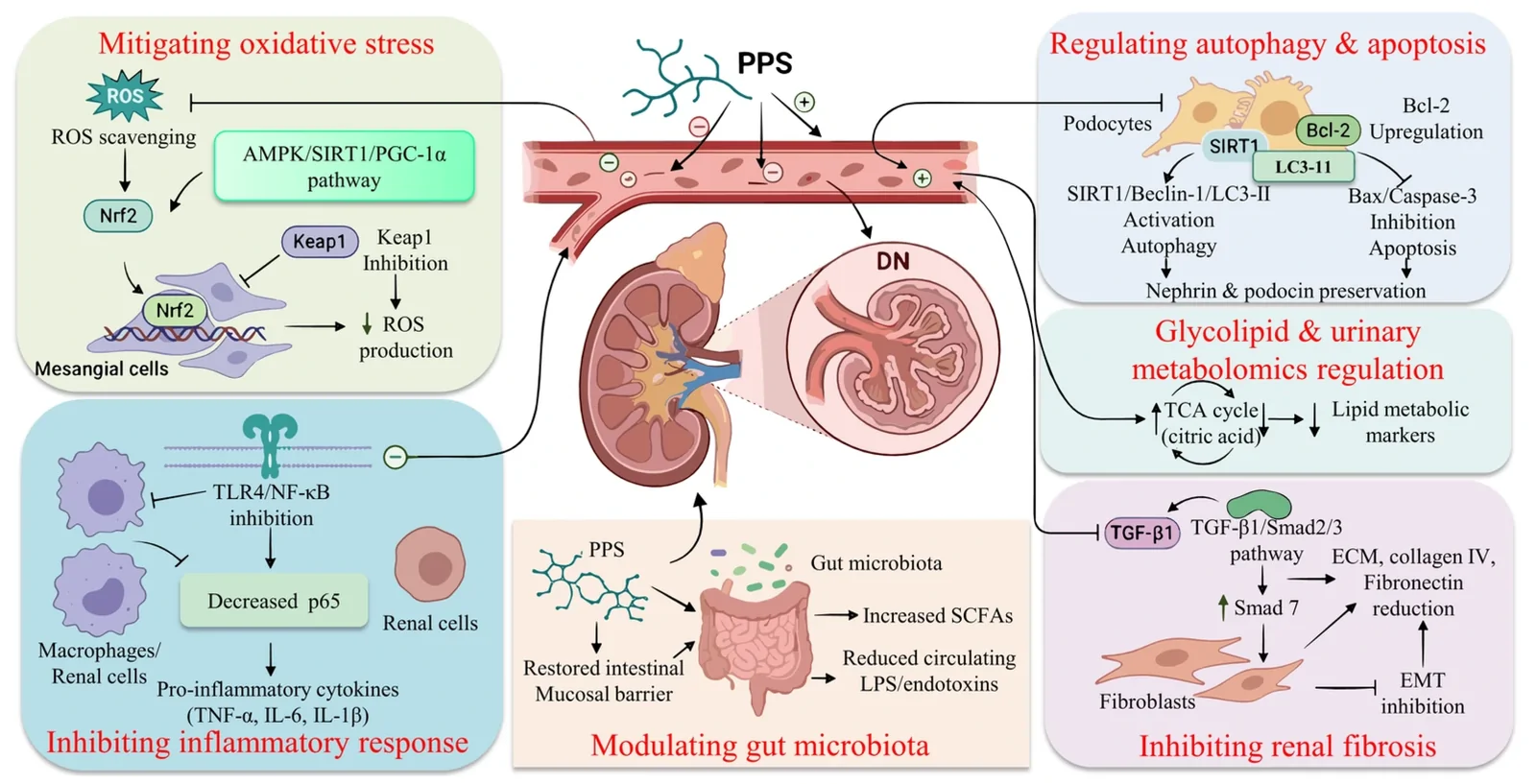
The mechanism by which PPS exert their anti-diabetic nephropathy effects.

The pathogenesis of DN is complex and involves multiple factors. Accumulating evidence from both in vivo and in vitro studies has demonstrated that PPS not only significantly reduce blood glucose levels and ameliorate insulin resistance but also improve renal function by lowering serum creatinine, blood urea nitrogen, and urinary microalbumin levels, thereby alleviating glomerular and tubular injury in DN mice [16,19,32]. However, these findings are predominantly derived from preclinical models, and their clinical relevance remains to be validated. This section mainly elaborates on the mechanisms by which PPS exert protective effects against DN from the perspectives of mitigating oxidative stress, inhibiting inflammatory responses, inhibiting renal fibrosis, regulating autophagy and modulating gut microbiota. The specific mechanisms by which PPS ameliorate DN are summarized in Figure 2.

### 3.1. Mitigate Oxidative Stress

Oxidative stress is considered one of the core mechanisms underlying the initiation and progression of DN. Chronic hyperglycemia promotes the excessive production of reactive oxygen species (ROS), leading to lipid peroxidation, protein damage, and DNA injury, which in turn trigger renal cellular dysfunction and death [30]. As natural antioxidants, PPS exert renoprotective effects via directly scavenging free radicals and indirectly activating the endogenous antioxidant system. Superoxide dismutase (SOD) and glutathione (GSH) eliminate ROS to maintain redox homeostasis, thereby conferring renal protective functions. Malondialdehyde (MDA), a major end product of lipid peroxidation, is regarded as a biomarker of oxidative stress [33]. Cumulative evidence has demonstrated that PPS may elevate the activities of antioxidant enzymes, including GSH, SOD, catalase (CAT) and glutathione peroxidase (GSH-px), in diabetic mice, while reducing MDA levels to alleviate oxidative-stress-induced renal injury [11,20,30].

In addition, long-term hyperglycemia can trigger cross-linking reactions between glucose and proteins, resulting in the massive formation of advanced glycation end-products (AGEs). AGEs bind to their specific cellular receptor (RAGE), subsequently activating oxidative-stress responses and stimulating the production of ROS [34]. Moutan cortex polysaccharides can attenuate AGE-induced ROS overproduction and reduce serum levels of AGEs and RAGE, thereby ameliorating renal injury in DN [12]. Furthermore, *Dipsacus asper* polysaccharides [16], *Liriope spicata* var. *prolifera* polysaccharides [20], and arabinose derived from *Angelica sinensis* [22] exert preventive or delaying effects on the progression of DN by disrupting the AGE–RAGE interaction.

The transcription factor Nrf2 is a vital signaling molecule that regulates antioxidant responses. It modulates a variety of intracellular antioxidants, phase II detoxifying enzymes and other functional proteins to scavenge diverse xenobiotics, thereby suppressing ROS and maintaining cellular homeostasis [35]. Under physiological conditions, Nrf2 remains in an inactive state. Upon oxidative-stress stimulation, however, Nrf2 translocates into the nucleus and activates a panel of antioxidant-related genes, which protects cells against oxidative injury [36,37]. For instance, *Astragalus mongholicus* polysaccharides markedly upregulate the expression of Nrf2, GCLC, NQO1, HO-1 and SOD, whereas they downregulate Keap-1 expression in mice with diabetic nephropathy [11]. Similarly, Burdock fructooligosaccharide [27] and *Momordica charantia* polysaccharides [24] have also been reported to exert antioxidant effects via the Nrf2/HO-1 signaling pathway. It is well known that the adenosine monophosphate-activated protein kinase (AMPK) signaling pathway exerts crucial functions in the regulation of lipid, glucose and energy metabolism. Activated AMPK signaling can mitigate oxidative stress and suppress cell apoptosis by upregulating the expression of silence information regulator T1 (Sirt1) and peroxisome proliferator-activated receptor γ coactivator (PGC-1α) [38]. Okra polysaccharides have been found to exert antioxidant effects by activating the Nrf2/HO1 and AMPK/Sirt1/PGC-1α signaling pathways [25].

### 3.2. Inhibit Inflammation

DN is characterized as a low-grade, persistent chronic inflammatory disease, in which hyperglycemia and oxidative-stress injury can continuously activate the systemic inflammatory response system. Following renal tissue damage, substantial amounts of pro-inflammatory cytokines, including tumor necrosis factor-α (TNF-α), interleukin-6 (IL-6), and interleukin-1β (IL-1β), are released, accompanied by the activation of classic inflammatory signaling pathways such as NF-κB and the Janus kinase/signal transducer and activator of transcription pathway. Persistent activation of these inflammatory pathways further promotes the secretion and infiltration of inflammatory cytokines, establishing a vicious cycle of oxidative stress–inflammatory activation–tissue injury. This cascade eventually triggers glomerular mesangial proliferation, tubulointerstitial inflammatory infiltration and renal microvascular damage, progressively aggravating renal dysfunction [39]. In vivo experiments have demonstrated that intervention with PPS is associated with reduced levels of inflammatory mediators, including TNF-α, IL-6, IL-1β, monocyte chemotactic protein 1, intercellular adhesion molecule 1, and vascular cell adhesion protein-1, in both serum and renal tissues of DN model animals [11,12,30,40].

TLR4 is a critical receptor that recognizes pathogen-associated molecular patterns and damage-associated molecular patterns (DAMPs), and its expression is markedly upregulated in DN [41]. As a downstream effector of the TLR4 signaling pathway, NF-κB is a ubiquitous and essential nuclear transcription factor involved in inflammatory responses. Under physiological homeostasis, NF-κB exists in the cytoplasm as an inactive heterodimer (p65) bound to the inhibitory protein IκBα to form a cytoplasmic complex. Upon activation of TLR4 signaling by external stimuli, IκBα kinase undergoes phosphorylation and subsequent degradation, allowing the NF-κB (p65) subunit to translocate from the cytoplasm into the nucleus, where it initiates the transcription of pro-inflammatory genes [42]. Accordingly, the TLR4/NF-κB signaling pathway serves as a critical axis that mediates immune-inflammatory responses and plays a vital role in the pathogenesis of DN. *Astragalus Mongholicus* polysaccharides [40], *Lycium barbarum* polysaccharides [32] and *Siraitia grosvenorii* polysaccharides [31] are capable of lowering the expression of inflammatory cytokines in kidney tissues via inhibition of TLR4/NF-κB pathway activation. Furthermore, HMGB1, as a DAMP molecule, can interact with TLR4 to activate NF-κB and promote the production of pro-inflammatory cytokines [43]. Liu et al. demonstrated that *Bupleurum* polysaccharides alleviate renal inflammatory injury in DN model mice by inhibiting the HMGB1-TLR4 signaling pathway [10].

### 3.3. Inhibit Renal Fibrosis

Renal fibrosis represents the core pathological alteration in the middle-to-late stage of DN, which is mainly characterized by excessive deposition of extracellular matrix (ECM). Transforming growth factor-β (TGF-β) is a key cytokine that modulates the progression of renal fibrosis by regulating cell proliferation and differentiation, as well as the synthesis and degradation of extracellular-matrix components [44]. Smad proteins constitute a family of signal-transducing molecules downstream of TGF-β1. During the pathogenesis of kidney diseases, Smad2 and Smad3 are recognized as the major effector proteins, whereas Smad7 exerts inhibitory effects on the TGF-β1 signaling pathway. In DN, TGF-β1 expression is markedly upregulated, which triggers the phosphorylation of Smad proteins and subsequently promotes the expression of fibrosis-related genes [45]. Numerous PPS have been shown to significantly downregulate the protein expression of TGF-β1 and p-Smad2/3 while upregulating the levels of the negative regulatory factor Smad7, thereby inhibiting glomerulosclerosis and tubulointerstitial fibrosis and delaying irreversible renal function impairment [11,18,30].

In addition, p-Smad2/3 translocate from the cytoplasm to the nucleus and act as transcription factors that regulate extracellular-matrix components such as fibronectin (FN) and type-IV collagen [46]. *Astragalus* polysaccharides [47] and *Bupleurum* polysaccharides [10] not only can suppress the activation of the TGF-β/Smad signaling pathway but also downregulate the expression levels of α-smooth-muscle actin, type-I collagen, type-III collagen, type-IV collagen and FN, thereby blocking the epithelial–mesenchymal transition (EMT) process and reducing ECM deposition. This multi-target action pattern represents a remarkable advantage of PPS over single-target chemical drugs. Nevertheless, several studies have demonstrated that low concentrations of TGF-β1 tend to activate the Smad-dependent pathway, whereas high concentrations potently trigger non-Smad pathways [48]. For instance, Huangjing polysaccharides alleviated renal fibrosis in DN via inhibiting the TGF-β1/PI3K/Akt signaling pathway [49].

Notably, *Dendrobium officinale* Kimura & Migo polysaccharide had been shown to inhibit high-glucose-induced fibrosis in renal tubular epithelial cells through modulation of the miRNA-34a-5p/SIRT1 signaling pathway [21]. Additionally, sea buckthorn berry polysaccharide attenuated renal fibrosis and suppressed the EMT process by downregulating the expression of matrix metalloproteinases (MMP2) and tissue inhibitors of metalloproteinases (TIMP2), as well as by blocking the p311/TGF-β1/Fstl1 pathway [29].

### 3.4. Regulate Cell Autophagy and Apoptosis

Podocyte injury and apoptosis represent critical events in the pathogenesis of proteinuria in DN. Nephrin and podocin are key structural proteins of the podocyte slit diaphragm, and their downregulation serves as an early marker of podocyte damage [50,51]. Astragalus polysaccharide [17] and *Pseudostellaria heterophylla* polysaccharide [19] have been shown to significantly upregulate the expression levels of nephrin and podocin in the glomeruli of DN model animals, thereby preserving the structural integrity of the slit diaphragm and, consequently, reducing proteinuria. Whether similar effects occur in patients with DN requires further investigation. Autophagy is a critical quality-control mechanism by which cells remove damaged organelles and aberrant proteins, playing an essential role in maintaining podocyte homeostasis. Silent information regulator 1 (SIRT1), a nicotinamide adenine dinucleotide-dependent histone deacetylase, acts as an intracellular energy sensor. Under diabetic nephropathy conditions, the expression level of SIRT1 is decreased in renal tissues [52]. *Pseudostellaria heterophylla* polysaccharide [19] and okra polysaccharide [25] have been shown to elevate SIRT1 expression levels and alleviate renal injury. Furthermore, SIRT1, acting as a positive regulator of autophagy, enhances autophagy through the deacetylation of autophagy-related proteins [53]. *Astragalus* polysaccharide has been reported to significantly reduce the protein expression of p62 and increase the LC3-II/LC3-I ratio, as well as Beclin-1 protein expression via activation of the SIRT1/FOXO3a/BNIP3 or SIRT1/FoxO1 pathways [17,54]. Using high-throughput RNA-sequencing technology, Chen et al. revealed that Astragalus polysaccharides could promote autophagy and alleviate diabetic nephropathy by targeting the lncRNA Gm41268/PRLR pathway [55].

Podocyte injury can be triggered by cellular apoptosis. High-glucose conditions, accumulation of ROS, and stimulation by inflammatory cytokines can induce disruption of the podocyte cytoskeletal architecture and aberrant expression of apoptosis-related proteins, leading to a reduction in podocyte number and impairment of podocyte function [56]. Burdock fructooligosaccharide [27] and okra polysaccharide [25] have been shown to reduce podocyte apoptosis by upregulating the expression level of Bcl-2, downregulating the expression of Bax, and inhibiting the activation of cleaved caspase-3.

### 3.5. Modulate Gut Microbiota

Accumulating evidence suggests that the gut microbiota may contribute to the initiation and progression of DN by affecting intestinal barrier function, renal metabolism and inflammation, as well as immune microenvironment homeostasis. As the largest microecosystem in the human body, the gut microbiota plays an important role in maintaining systemic metabolic homeostasis [57]. Studies have demonstrated that patients with DN exhibit a reduced glomerular filtration rate, which causes massive accumulation of metabolic wastes that cannot be excreted via the kidneys, thereby aggravating gut dysbiosis, characterized by decreased abundance of beneficial bacteria and overgrowth of opportunistic pathogens. Gut dysbiosis can impair intestinal barrier integrity, resulting in increased translocation of endotoxins (lipopolysaccharide) into the circulation and subsequent activation of systemic chronic inflammatory responses. Furthermore, gut-microbiota-derived metabolites, such as trimethylamine N-oxide, have been shown to promote tubulointerstitial fibrosis [58].

As natural prebiotics, PPS exert renoprotective effects through modulation of the gut microbiota. *Astragalus mongholicus* polysaccharide has been shown to significantly increase the abundance of *Muribaculaceae*, *Ruminococcus_1*, *Lachnoclostridium* and *Phascolarctobacterium* in DN rats [11]. Moutan cortex polysaccharide significantly elevated the abundance of *Akkermansia* and *Lactobacillus*, while it reduced the proportion of harmful bacteria [9]. These changes were closely associated with elevated levels of short-chain fatty acids, reduced serum inflammatory cytokines, and improved renal function. Blackcurrant polysaccharide, through enrichment of *Akkermansia muciniphila*, increased serum levels of glycoursodeoxycholic acid and activated the renal GPBAR1 signaling pathway, thereby inhibiting the NF-κB/NLRP3 inflammatory pathway and TGF-β-mediated fibrosis [15]. In addition, *Bupleurum* polysaccharides was found to enhance microbial species diversity and richness, restructure the gut microbial community in mice, lengthen ileal villi, alleviate aberrant colonic cell proliferation, and improve intestinal barrier function. Meanwhile, it reversed gut dysbiosis in DN-model mice mainly by boosting the abundance of butyrate-producing bacteria [59].

In recent years, the gut–kidney axis theory has provided a novel perspective for understanding the pathogenesis, prevention and treatment of DN. Nevertheless, studies investigating the effects of PPS on renal injury in DN via gut-microbiota-derived metabolites remain relatively scarce and are still in their infancy. At present, most existing studies lack detailed analyses on the abundance and proliferative capacity of bacteria responsible for producing gut microbial metabolites. Therefore, the specific mechanisms by which plant polysaccharides intervene in DN through gut microbiota modulation warrant further in-depth investigation.

It is worth noting that most of the abovementioned protective mechanisms of plant polysaccharides, including antioxidative, anti-inflammatory, anti-fibrotic, autophagy-regulating and gut-microbiota-modulating effects, are not uniquely specific to DN. These biological pathways are also implicated in other renal disorders, as well as metabolic and hepatic diseases. Nevertheless, the initiating pathological stimuli differ across disease contexts. In DN, sustained hyperglycemia drives excessive ROS generation and AGE accumulation, rendering the AGE–RAGE cascade particularly prominent, which distinguishes DN from non-diabetic kidney injury. Therefore, although effector pathways overlap, their relative contribution varies among pathological conditions.

In addition to the abovementioned main mechanisms, PPS can ameliorate renal injury in DN by regulating glycolipid metabolism and urinary metabolites. After four weeks of intervention with black onion polysaccharide, serum levels of triglycerides (TGs), total cholesterol, and low-density lipoprotein cholesterol were significantly reduced in db/db mice [14]. In addition to markedly decreasing TG levels, *Polygonati rhizoma* polysaccharides also significantly elevated high-density lipoprotein levels [18]. Bai et al., employing urinary metabolomics, demonstrated that red kidney bean polysaccharides and small black soybean polysaccharides exerted renoprotective effects against DN by regulating key urinary metabolites involved in the tricarboxylic-acid cycle and phenylalanine metabolism, such as citric acid and cis-aconitic acid [28]. A urinary metabolomics study on rats treated with fermented celery polysaccharides demonstrated that its protective effects were mainly enriched in amino-acid-metabolism- and antioxidant-related metabolic pathways [23].

## 4. Structure–Activity Relationship

The biological activity of PPS is closely related to their structural characteristics, but the study of the structure–activity relationship is still at an early stage. Available evidence indicates that molecular weight, monosaccharide composition, glycosidic-bond type and branching degree may affect the anti-DN activity of polysaccharides. Molecular weight constitutes a critical factor influencing the bioactivity of polysaccharides. A moderate molecular weight may facilitate the binding of polysaccharides to their targets, as well as their absorption and utilization in vivo. Nevertheless, there is no simple linear relationship between molecular weight and bioactivity, and polysaccharides from different sources may possess distinct optimal molecular-weight ranges [60]. Monosaccharide composition determines the basic structural backbone of polysaccharides. PPS are predominantly rich in glucose and galactose; however, the specific monosaccharide composition that is most favorable for anti-DN activity remains inconclusive. Feng et al. extracted two crude polysaccharides from *Bupleurum*, designated as BCP and BPs, both of which contained three fractions with different molecular weights. The molecular weights of BCP were 2,917,731 Da; 281,670 Da; and 2707 Da, whereas those of BPs were 2,765,184 Da; 182,602 Da; and 32,877 Da. Furthermore, both polysaccharides shared the same monosaccharide composition, consisting of Man, Rha, GlcA, GalA, Glc, Gal, Xyl, and Ara but differed in their molar ratios. The results from DN animal experiments demonstrated that BCP possesses potent anti-inflammatory activity and gut-microbiota-regulatory capacity, which may be attributed to its higher contents of Glc and Gal [59].

Glycosidic-bond types and branching degree determine polysaccharide higher-order structures, and strongly influence digestibility and molecular recognition by host receptors or gut microbes. Starch is a plant-derived α-D-glucan polymer connected by α-1,4 and α-1,6 glycosidic bonds and is chemically classified as a plant polysaccharide. Ordinary starch is rapidly hydrolyzed by gastrointestinal α-amylase and absorbed as glucose, with limited capacity to reach the colon or exert direct renoprotective effects in DN. This is why de-starch steps are commonly applied during the preparation of bioactive plant polysaccharides to eliminate starch interference. Notably, resistant starch escapes small-intestinal digestion and can act as prebiotics to ameliorate metabolic disturbance indirectly via gut microbiota, but this effect is distinct from the direct renal-targeting activity shown by most bioactive β-linked heteropolysaccharides. The ratio of α-glycosidic to β-glycosidic bonds, as well as the structural features of the main chain and side chains, may influence the interaction between polysaccharides and receptor proteins. The main chain of Moutan cortex polysaccharide is composed of →4)-α-D-Glcp-(1→, 5-α-L-Araf-(1→, and 3,5-α-L-Araf-(1→, with branches at the O-3 position. This structural characteristics may be correlated with its anti-inflammatory and anti-fibrotic activities [12].

Overall, the current understanding of the structure–activity relationship of PPS against DN remains very limited, and more systematic studies on structural characterization and bioactivity evaluation are urgently required.

## 5. Conclusions and Future Perspectives

DN is one of the most common and severe microvascular complications of diabetes mellitus, characterized by a complex pathogenesis and great clinical-treatment challenges. With unique advantages, including abundant sources, high biosafety, diverse biological activities and multi-target synergistic intervention, PPS have shown promising renoprotective effects in preclinical models and may represent potential adjunctive strategies for DN. However, there are still many challenges in the clinical transformation of PPS. It should be critically noted that nearly all existing evidence for PPS against DN originates from cell-culture and preclinical animal studies; high-quality, large-sample-size human clinical trials remain scarce. Many published animal investigations utilize crude polysaccharide preparations with variable purity, which raises concerns about reproducibility. Up to now, robust clinical evidence supporting direct renoprotection of plant polysaccharides in DN patients is lacking. Heterogeneity of botanical raw materials, undefined polysaccharide structures, absence of unified quality-control specifications, and insufficient long-term human safety data constitute major obstacles for clinical translation. Firstly, the advanced structure of polysaccharides is difficult to analyze, and the structure–activity relationships are not yet well defined, which hinders the optimization of drugs based on structure. Secondly, the oral bioavailability of polysaccharides is generally low, and the absorption, distribution, metabolism and excretion patterns in vivo remain poorly understood. Thirdly, the quality-control standards for polysaccharide drugs are not yet perfect, and the activity of different batches of products may vary. Furthermore, the current extraction of PPS predominantly relies on conventional water extraction and alcohol precipitation methods, which are accompanied by drawbacks including low extraction yield, unsatisfactory purity, high impurity content and severe loss of active components. Meanwhile, research on polysaccharide preparations lags behind. Most existing studies are still performed using crude polysaccharide solutions for administration, and the development of novel preparations such as tablets, capsules and nano-formulations is lacking. The inherent instability, low bioavailability, and insufficient targeting of polysaccharides pose prominent challenges, making it difficult to meet the demands of clinical application and industrial translation.

To address the abovementioned issues, future research should focus on the following directions: (1) advanced analytical techniques (e.g., NMR, mass spectrometry, and atomic force microscopy) should be employed to thoroughly elucidate the high-order structures of polysaccharides and construct a systematic database for structure–activity relationships; (2) further investigations into the interactions between polysaccharides and gut microbiota should be carried out to clarify the mechanism underlying the “polysaccharide–gut microbiota–metabolite–kidney” axis; (3) novel delivery systems (such as nanoparticles and liposomes) should be developed to improve the oral bioavailability and targeting ability of polysaccharides; (4) rigorously designed preclinical and clinical studies should be performed to systematically evaluate the safety and efficacy of plant polysaccharides; (5) artificial-intelligence technologies including machine learning and deep learning should be utilized to establish prediction models for polysaccharide structure–activity relationships to accelerate the screening and optimization of bioactive polysaccharides.

The research on PPS for the prevention of DN is currently at a critical stage of transitioning from the laboratory to clinical application. With advances in analytical technologies and the deep understanding of the gut–kidney axis, PPS are expected to become an important component in comprehensive therapeutic strategies for DN, providing safer and more effective treatment options for patients.

## Data Availability

No new data were created or analyzed in this study. Data Sharing is not applicable to this article.
